# ESC, ALK, HOT and LOT: Three Letter Acronyms of Emerging Renal Entities Knocking on the Door of the WHO Classification

**DOI:** 10.3390/cancers12010168

**Published:** 2020-01-09

**Authors:** Farshid Siadat, Kiril Trpkov

**Affiliations:** Department of Pathology and Laboratory Medicine, University of Calgary and Alberta Precision Laboratories, Rockyview General Hospital, 7007 14 Street, Calgary, AB T2V 1P9, Canada; Farshid.Siadat@albertaprecisionlabs.ca

**Keywords:** kidney, emerging entity, new entity, oncocytic renal tumor, unclassified renal cell carcinoma, unclassified renal tumor, anaplastic lymphoma kinase rearrangement, ALK, ESC, HOT, LOT

## Abstract

Kidney neoplasms are among the most heterogeneous and diverse tumors. Continuous advancement of this field is reflected in the emergence of new tumour entities and an increased recognition of the expanding morphologic, immunohistochemical, molecular, epidemiologic and clinical spectrum of renal tumors. Most recent advances after the 2016 World Health Organization (WHO) classification of renal cell tumors have provided new evidence on some emerging entities, such as anaplastic lymphoma kinase rearrangement-associated RCC (ALK-RCC), which has already been included in the WHO 2016 classification as a provisional entity. Additionally, several previously unrecognized entities, not currently included in the WHO classification, have also been introduced, such as eosinophilic solid and cystic renal cell carcinoma (ESC RCC), low-grade oncocytic renal tumor (LOT) and high-grade oncocytic renal tumor (HOT) of kidney. Although pathologists play a crucial role in the recognition and classification of these new tumor entities and are at the forefront of the efforts to characterize them, the awareness and the acceptance of these entities among clinicians will ultimately translate into more nuanced management and improved prognostication for individual patients. In this review, we summarise the current knowledge and the novel data on these emerging renal entities, with an aim to promote their increased diagnostic recognition and better characterization, and to facilitate further studies that will hopefully lead to their formal recognition and consideration in the future classifications of kidney tumors.

## 1. Eosinophilic Solid and Cystic Renal Cell Carcinoma (ESC RCC) 

Eosinophilic solid and cystic renal cell carcinoma (ESC RCC) has been recently characterized as an emerging renal entity that exhibits a set of well-defined clinical, pathological, immunohistochemical and molecular features [1,2,3]. Because it was described very recently, it is not yet included in the 2016 WHO classification of genitourinary tumors [4]. These types of tumors have likely been previously designated as “unclassified renal cell carcinoma” or “unclassified renal neoplasm (or renal cell carcinoma) with oncocytic or eosinophilic morphology”. Great majority of ESC RCC are sporadic and are found in non-syndromic setting, although a subset of identical tumors have been documented in patients with a tuberous sclerosis complex (TSC) [5,6]. The great majority of ESC RCC are small, solitary tumors of low stage that are found in female patients, and generally exhibit indolent behaviour, although rare cases have been documented with metastatic disease [1,2,7,8,9,10]. The most salient features of ESC RCC and the other emerging entities included in this review are summarized in Table 1.

### 1.1. Clinical Features

ESC RCC are typically sporadic tumors, not associated with TSC; however, about 10% or less of these tumors with virtually identical features have been documented in TSC patients [1,2,3,5]. ESC RCC are typically solitary, unifocal tumors of small size and low stage (mostly pT1, rarely pT2 or pT3) [1,2,3,11]. Occasional multifocal and bilateral cases have also been reported [2,8,12]. The patients are overwhelmingly females, and only rare cases have been reported in males. In an unselected patient cohort, the median age was 55 years, with a broad patient age range (32–79 years) [1,2]. However, a recent study of previously ‘unclassified’ eosinophilic renal tumors in younger patients (defined as age 35 years or younger), found 10 ESC RCC, which represented 30% of this cohort, with a median age of 27 years [12]. The true incidence of this tumor is currently unknown.

Although the great majority of ESC RCC exhibit indolent behavior, four cases have so far been reported with metastases, which justifies the “RCC” designation for this entity and reiterates the need for an ongoing clinical surveillance for these patients [1,2,7,8,9]. Additional studies are needed to fully determine the biologic behavior of ESC RCC, because to date, there are only few well-documented series with sufficient follow-up [1,2,10].

### 1.2. Pathological Features

#### 1.2.1. Gross Features

**Figure 1:**
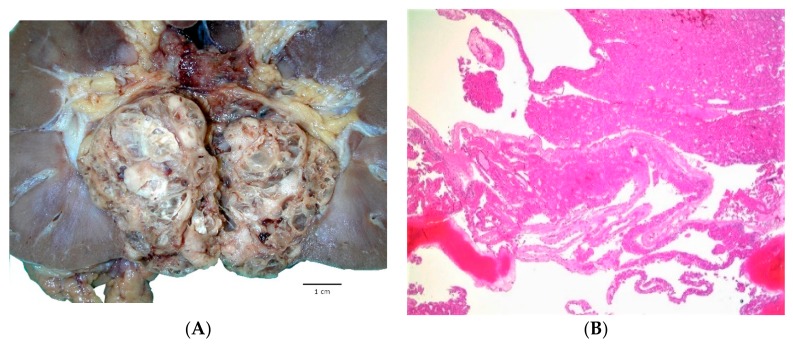

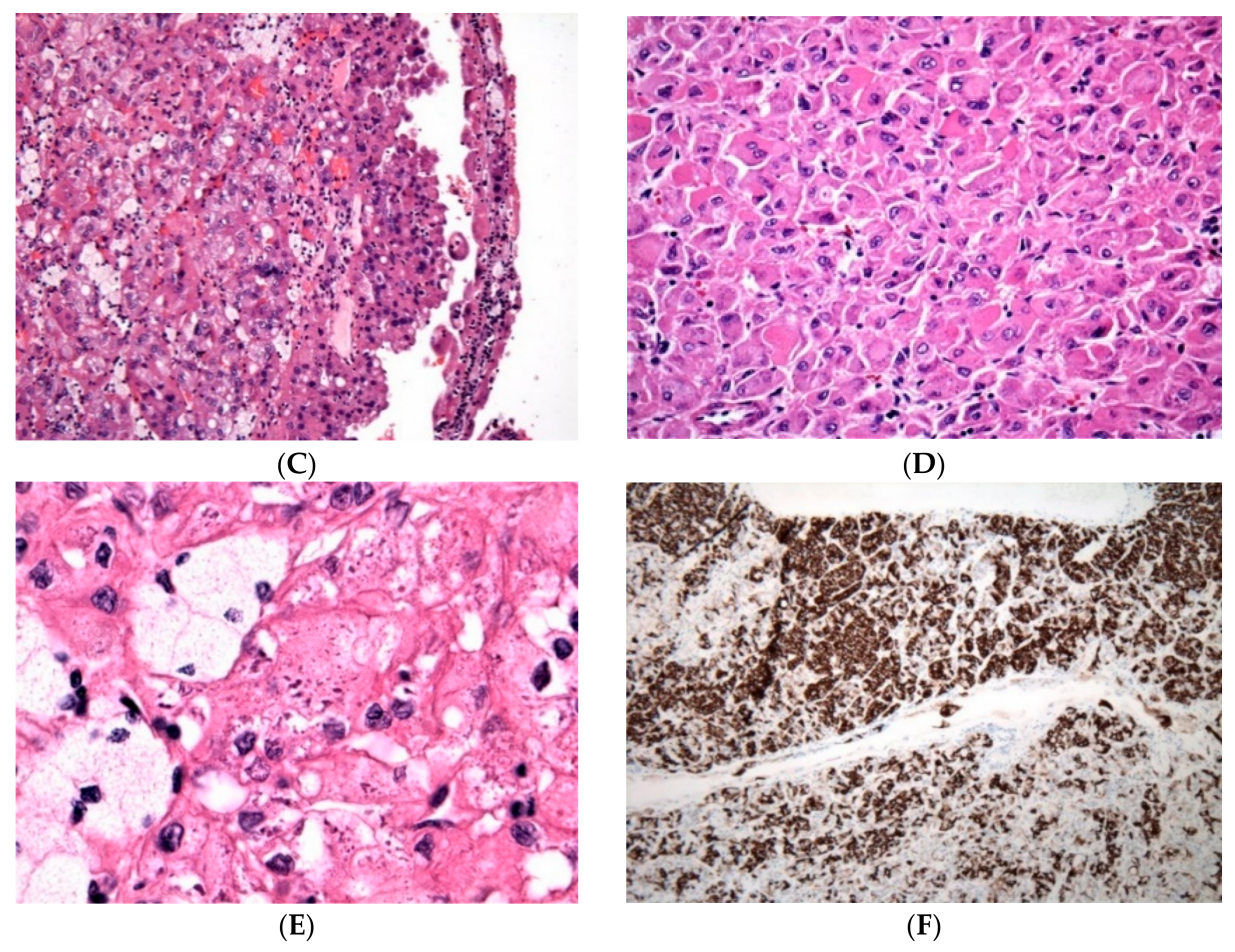
Eosinophilic solid and cystic renal cell carcinoma (ESC RCC) show grossly mixed macrocystic and solid appearances (**A**). Solid and cystic areas can also be appreciated at low power (**B**). The solid areas demonstrate diffuse and compact growth with adjacent cyst trabeculae showing hobnailing (**C**). Eosinophilic cells exhibit voluminous cytoplasm with readily recognizable coarse cytoplasmic granules (‘stippling’); aggregates of foamy histiocytes and lymphocytes are often found (**D**,**E**). CK20 is positive in the majority of ESC RCC (**F**).

The proposed name of this tumor entails the designations “solid and cystic” which succinctly conveys the main gross features [1,2,3,4,5]. ESC RCC are well-delineated tumors, showing mixed macrocystic and solid patterns (Figure 1A) [1,2]. The cysts are often multifocal and vary in size from few millimeters to few centimeters; the presence of macrocysts is one of the key gross features. Rare cases demonstrated almost exclusively solid growth with only rare identifiable microscopic cysts [1,2]. The tumor cut surface is yellow/gray/tan. Most of the reported tumors were smaller than 5 cm (median: 3.1 cm, mean 4.2 cm), but the reported tumor size was variable, from 1.2 to as 13.5 cm in greatest dimension, based on the largest series by Trpkov et al. [1,2].

#### 1.2.2. Microscopic Features

ESC RCC are circumscribed tumors, but without a well-formed capsule at the periphery.

The epithelial lining of the cysts has a hobnail arrangement and the cyst trabeculae (i.e., the solid parts between the cysts) can vary in thickness (Figure 1B,C) [1,2]. The solid tumor areas have identical appearances as seen in the trabeculae between the cysts, showing diffuse, compact acinar or compact nested growth (Figure 1D). Scattered aggregates of foamy histiocytes and lymphocytes are often found (Figure 1C,E). The cells typically show eosinophilic, voluminous cytoplasm, with readily identifiable coarse, basophilic to purple, cytoplasmic granules (‘stippling’), which represents a very helpful morphologic feature; these granules correspond to aggregates of rough endoplasmic reticulum, observed on electron microscopy (Figure 1E) [1]. The nuclei are round to oval with focally prominent nucleoli, equivalent to WHO/ISUP grade 2 or 3 (Figure 1D,E). Focal nuclear irregularities can also be seen.

Other less common or less specific features can also be present, including focal papillary growth, focal “clear cell” areas, as well as focal insular or tubular growth and clusters of multinucleated cells [1,2]. Cell size variations or architectural pattern variations can also be observed within individual cases. Intracytoplasmic vacuolization, either microvesicular or macrovesicular, is also common and psammoma bodies can be found in about half of the cases [1,2].

#### 1.2.3. Immunohistochemical and Molecular Features

Immunohistochemistry (IHC) for CK20 is positive in great majority of ESC RCC (Figure 1F), either as diffuse or focal reactivity, and it is usually paired with a negative or only focally positive CK7 (in about a quarter of cases) [1,2]. Of note, negative CK20 can also be observed in about 10%–15% of otherwise typical cases, together with either negative or focally positive CK7 [1,2,3]. To our knowledge, none of the reported ESC RCC cases so far demonstrated a CK20 negative/CK7 positive immunophenotype, which may be helpful in differentiating ESC RCC from other eosinophilic renal tumors. Other positive stains also include PAX8, AE1/AE3, CK8/18 and vimentin; rare cases can be negative or only focally positive for cytokeratin AE1/AE3 [1,2]. Cathepsin K is also reactive in great majority of ESC RCC, either as diffuse or focal positivity (personal unpublished observations), in line with some of the reported observations of cathepsin K reactivity in ESC RCC [10,12]. Negative stains include CD117, CA9, HMB45 and melan A [1,2].

The molecular characterization of ESC RCC by next generation sequencing (NGS) revealed recurrent and mutually exclusive somatic bi-allelic loss or mutations in of TSC gene family, including *TSC2* and *TSC1* in 85% (6/7) of evaluated cases [13]. Parilla et al. characterized two cases of sporadic ESC RCC in patients without clinical features of tuberous sclerosis, which demonstrated pathogenic somatic TSC2 gene mutations [9]. These mutations were without other alterations in any other genes associated with RCC, suggesting that sporadic ESC RCC may be characterized by somatic tuberous sclerosis gene mutations (TSC2) [9]. Palsgrove and al have also confirmed a consistent presence of either TSC1 or TSC2 gene mutations in pediatric ESC RCC (8/9 cases) and adult ESC RCC (6/6 cases). These included a metastatic ESC RCC which had a complete response to mTOR targeted therapy [10]. Molecular karyotype profiling of ESC RCC has also shown common and recurring genomic changes, including copy number (CN) gains at 16p13-16q23, 7p21-7q36, 13q14, and 19p12 and CN losses at Xp11.21 and 22q11 [2]. Loss of heterozygosity (LOH) alterations were identified at 16p11.2-11.1, Xq11-13, Xq13-21, 11p11, 9q21-22, and 9q33 [2]. Many of the genes and gene sets in the affected regions are involved in the regulation of *MTOR* signaling pathway and indicate that ESC RCC genomic alterations are different from those found in the currently recognized renal neoplasms [2,9,10]. Although these molecular alterations are neither pathognomonic nor specific for ESC RCC, taken together with the morphologic and immunohistochemical features seen in ESC RCC, signify a relatively compact and distinct morpho-molecular entity.

### 1.3. Differential Diagnosis

The wide spectrum of the renal tumours with eosinophilic cells should all be considered in the differential diagnosis of ESC RCC, which includes primarily renal oncocytoma and the eosinophilic variant of chromophobe renal cell carcinoma (Chr RCC), as well as some less common entities, such as succinate dehydrogenase (SDH)-deficient RCC, MiTF translocation RCC (particularly TFEB), and epithelioid angiomyolipoma (AML), as shown in Table 2. However, the morphologic features of ESC RCC observed on H&E and its immunohistochemical profile are generally sufficient for the diagnosis.

## 2. ALK Rearrangement-Associated RCC (ALK-RCC)

ALK rearrangement-associated RCC (ALK-RCC) is another novel renal entity that has already been included in the 2016 WHO classification as an “emerging/provisional” entity [3,4]. It encompasses renal carcinomas demonstrating translocation that result in a fusion of the ALK gene with various gene partners. ALK is located at 2p23 and it is a member of the receptor tyrosine kinase family and the insulin receptor superfamily [14]. Chromosomal rearrangements resulting in ALK fusion with several partner genes lead to aberrant ALK activation through the formation of oncogenic chimeric proteins. However, rearrangement of ALK is not restricted only to ALK-RCC, but has also been previously documented in other non-renal tumors, such as non-small-cell lung adenocarcinoma, anaplastic thyroid carcinoma, anaplastic large-cell lymphoma, and others [15]. Since the first report in 2011, less than 30 cases of ALK-RCC have been reported [16,17,18,19,20,21,22,23,24,25,26,27,28,29,30,31,32]. These tumors demonstrate considerable morphologic diversity, but their underlying commonality includes the ALK rearrangement that can be documented either by IHC or by molecular studies, such as fluorescence in situ hybridisation (FISH).

### 2.1. Clinical Features

ALK RCC are solitary tumors, typically not associated with any clinical syndromes. They are documented in patients with diverse racial background, including African American, Caucasian and Asian patients. However, a large study of 1019 kidney tumors in adult Polish patient cohort failed to identify a single ALK-RCC by using three different clones of anti-ALK antibodies for immunohistochemical evaluation [33].

ALK-RCC occurs slightly more often in male patients (M:F = 1.5:1) and has been documented in patients of wide age range, with clustering in children and adolescents (range, 3–19 years), younger and middle aged adults (range, 30–49 years), and in patients older than 50 years (range, 52–85 years). Some pediatric cases demonstrating *VCL–ALK* and *TPM3–ALK* fusions have been documented in African American patients with the sickle-cell trait [19,27,28].

Although the clinical behavior and the histopathologic characteristics of this tumor have not been fully established, they may exhibit adverse prognosis, including metastatic disease and death, which has been documented in about 30% of the cases, clearly substantiating their malignant potential [16,24,32]. The interest in this tumor has also been fortified by the availability of targeted therapies, such as ALK inhibitors alectinib and crizotinib in tumors demonstrating ALK rearrangement. A recent report described three patients with “metastatic ALK-rearranged papillary RCC” with documented ALK fusion (all with *EML4-ALK*), who were treated with alectinib, and all patients showed a demonstrable short term clinical and radiographic response [32]. Based on the provided data in the report, these three case likely represent types of tumors within the spectrum of the reported ALK-RCC [32].

### 2.2. Pathological Features

#### 2.2.1. Gross Features

**Figure 2:**
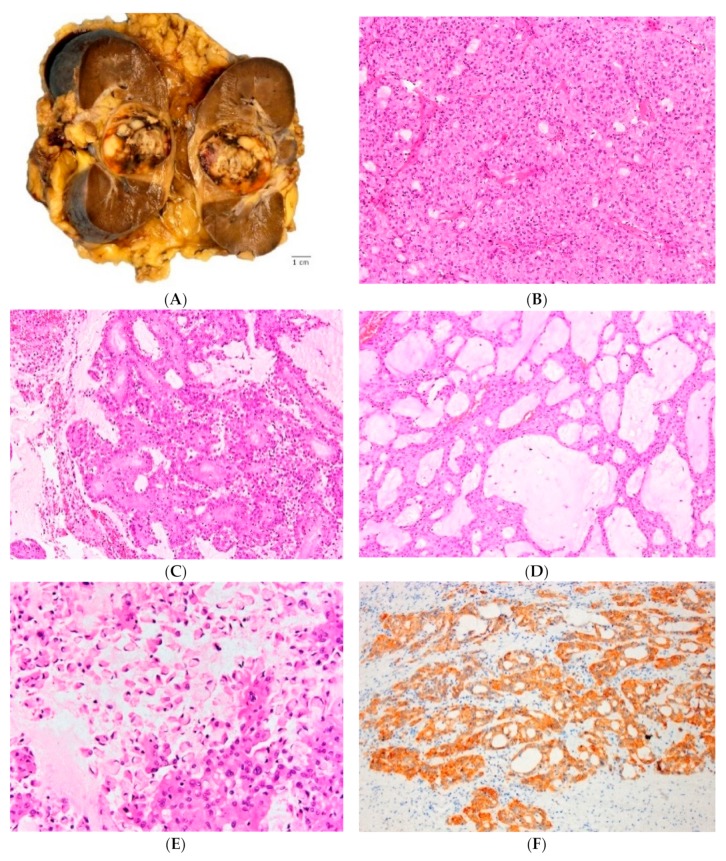
ALK rearrangement-associated RCC (ALK-RCC) shows a variegated gross appearance (**A**). On microscopy, multiple growth patterns can be seen in a single tumor, including solid (**B**), papillary (**C**), tubular and tubulocystic (**D**). Intracytoplasmic vacuoles and individual signet ring cells can also be found (**E**) often with a mucinous background (**D**,**E**). There is ALK1 protein expression on immunohistochemistry (**F**).

ALK-RCC are solitary, solid or solid–cystic tumors that show white-grey to yellow and variegated cut surface and range in size from 3 to 7 cm (Figure 2A). Most of the reported pediatric cases with *VCL-ALK* fusion were located in the medulla or the renal pelvis, while the remaining cases were mostly located in the renal cortex. Grossly identifiable necrosis and hemorrhage were also noted in some cases.

#### 2.2.2. Microscopic Features

Pediatric cases demonstrating *VCL–ALK* and *TPM3–ALK* fusions exhibited morphologic similarities to adult renal medullary carcinoma and collecting duct carcinoma, such as diffuse solid or reticular/syncytial/tubular growth, with a background of prominent and delicate vascular network, often admixed with significant lymphoplasmacytic inflammatory infiltrate and stromal desmoplasia [19,20,21,27,28]. The neoplastic cells in these pediatric cases were typically discohesive and showed variable cytomorphologies, including polygonal, spindle, cuboidal and low columnar. They also exhibited voluminous eosinophilic cytoplasm with common intracytoplasmic lumina; the nuclei were round to oval with focally prominent nucleoli.

The remaining types of ALK-RCC showed variable and complex morphologies, typically demonstrating multiple growth patterns in a single case, including solid, tubular or tubulo-cystic, papillary (or pseudopapillary), cribriform, trabecular, and signet-ring individual cell growth (Figure 2B–E). An extensive mucinous background, as well as intracytoplasmic mucin have been frequently found in these cases and may be a helpful clue for the diagnosis [16,18,23]. The cells had eosinophilic cytoplasm and showed variable morphologies, including rhabdoid, vacuolated, pleomorphic giant cell and small cell (metanephric adenoma-like) morphology. Occasional psammoma bodies were also present and coagulative necrosis and mitoses have also been found in these cases [16,24].

#### 2.2.3. Immunohistochemistry, Electron Microscopy and Molecular Features

Patients with ALK-RCC demonstrate diffuse cytoplasmic and membranous ALK protein expression by IHC, which can aid in screening suspicious cases (Figure 2F). However, for a definitive diagnosis, an ALK-rearrangement by FISH is necessary, typically showing an ALK split signal in the majority of tumor cells. ALK-RCCs are usually reactive for CK7, 34βE12, AMACR and vimentin; INI-1 expression is retained and Ki67 proliferation index is relatively low [19]. Melanocytic markers such as melan A, S100, HMB45, and cathepsin K are negative [16]. Immunoreactivity for TFE3, but without genuine TFE3-rearrangement by FISH, has been reported in some pediatric cases [19,20,21,25] and in one adult patient with ALK-RCC showing *TPM3-ALK* fusion [18].

Electron microscopy of ALK-RCC demonstrated the presence of microvilli, tight desmosomes, numerous lipofuscin-like structures, and a well-developed cytoskeleton in the cytoplasm [21,25,27].

Genetic studies have identified, so far, several ALK fusion gene partners, but in some cases none of these partners were found, which indicates the possibility that some additional gene partners have yet to be discovered. Until recently, *VCL-ALK* has been documented only in pediatric patients of African American descent with the sickle-cell trait [19,21,27,28]. However, a recent report identified *VCL-ALK* fusion in a 57 year-old Chinese woman, without any evidence of the sickle cell trait [31]. Four other ALK fusion gene partners have been also identified, both in pediatric and adult patients: *TPM3-ALK, EML4-ALK,* striatin *(STRN)-ALK,* and *HOOK1-ALK* [16,17,18,20,21,22,23]. Most recently, in a multi-institutional study of 12 novel ALK-RCC, we identified three new, previously unreported ALK gene partners: *CLIP1*, *KIF5B* and *KIAA1217* (data presented in part at the United States Canadian Academy of Pathology Annual Meeting 2019) [34].

### 2.3. Differential Diagnosis

The differential diagnosis of ALK-RCC is quite broad, primarily due to the presence of variable and diverse morphologies that can be observed in ALK-RCC, which may mimic other renal entities, such as renal medullary carcinoma (in children and adolescents), collective duct carcinoma, papillary RCC, MiTF RCC (TFE3 and TFEB), mucinous tubular and spindle cell carcinoma, and thyroid-like follicular RCC. However, ALK-RCC usually shows multiple, admixed growth patterns, often set in a mucinous background, generally demonstrates lesser degree of cytologic atypia, lack significant stromal desmoplasia and are non-infiltrative. ALK positive IHC or ALK rearrangements demonstrated by FISH are crucial in establishing the diagnosis. A notable pitfall in misdiagnosing ALK RCC as Xp11.2 translocation RCC may be due to its positivity for TFE3 by IHC in some cases; however, TFE3 rearrangement by FISH is lacking in these cases [19,20,25,27,28].

## 3. High-Grade Oncocytic Tumor (HOT) of Kidney

High-grade oncocytic tumor (HOT) is an entity with unique and readily recognizable morphology, composed predominantly of oncocytic cells with high-grade nuclei, prominent intracytoplasmic vacuoles, and demonstrates a relatively consistent IHC profile. HOT of kidney has recently been proposed [35] to represent a previously unrecognized and potentially new renal entity that is currently not listed in the 2016 WHO classification [4]. The first published study on this tumor in 2018 by He et al. described a multi-institutional series of 14 tumors, designated as “HOT” of kidney [35]. Soon thereafter, Chen et al. reported a single-institutional series of seven morphologically identical tumors that they designated “sporadic renal cell carcinomas with eosinophilic and vacuolated cytoplasm” [36]. Most recently, an example of an identical type of tumor was reported by Trpkov et al. in a patient with a Tuberous Sclerosis Complex (TSC) [37], in contrast to the sporadic cases included in the initial two series [35,36]. The tumors designated as “HOT” and “sporadic renal cell carcinomas with eosinophilic and vacuolated cytoplasm”, in our view, represent the same entity. In this review, we will use the designation “HOT” for this novel tumor, which we proposed initially as a provisional name for this entity [35]. The term “sporadic renal cell carcinomas with eosinophilic and vacuolated cytoplasm” used by Chen et al. despite being somewhat long, inaccurately stipulates that this tumor is a “carcinoma” prima-facie, without providing either evidence or justification for this terminology [36]. Importantly, the case reported in a TSC patient also argues against the use of the descriptor “sporadic” for these tumors [37].

HOT of kidney emerged from the spectrum of oncocytic (or eosinophilic) tumours that include renal oncocytoma, chromophobe renal cell carcinoma (Chr RCC), as well as previously reported tumors with “hybrid” morphology of oncocytoma-Chr RCC, either as sporadic “hybrid-oncocytic” tumors, or tumors found in a syndromic setting, such as Birt–Hogg–Dubé (BHD) syndrome, renal oncocytosis and TSC [5,38,39,40,41,42]. Indeed, it is not uncommon to encounter oncocytic tumors with mixed and unusual morphologies either in a syndromic or sporadic setting that are not easy to classify because they do not fit into any of the currently recognized renal tumor categories [38].

### 3.1. Clinical Features

All patients included in the He et al. and Chen et al. studies presented with non-syndromic, solitary tumors. HOT was found more often in females (M:F = 1:2.5), with median age of 50 and 55 years respectively, within a broad age range (range, 25 to 73 years) [35,36]. During the documented follow-up, all tumors demonstrated an indolent behavior, and although the follow-up was relatively limited in both series, some cases had a follow-up of more than 10 years. The prevalence of this type of tumor is currently unknown.

Of note, the one renal HOT reported in a TSC patient, was found in a 48 year old female with a known TSC, who had bilateral renal masses on routine ultrasound screening [37]. The tumor measured 1.3 cm and represented a solid, tan-brown neoplasm. Multiple small adjacent AML tumorlets were also found, as well as a small renal cell carcinoma with angioleiomyomatous (or smooth muscle) stroma, a type of renal neoplasm documented in a setting of TSC [5].

### 3.2. Pathological Features

#### 3.2.1. Gross Features

**Figure 3:**
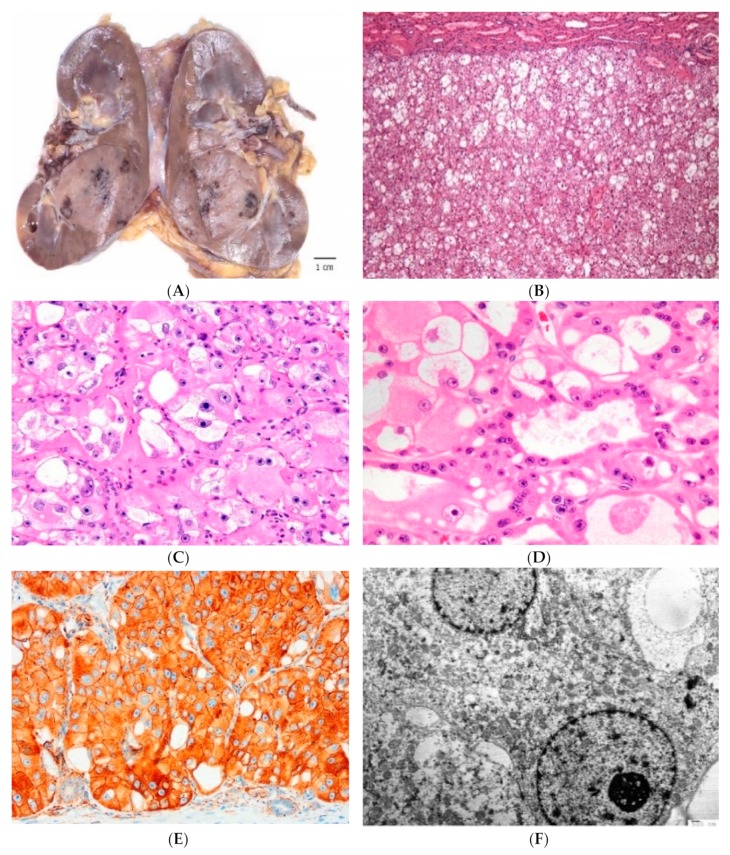
High-grade Oncocytic Tumor (HOT) of kidney often mimics the color of the adjacent renal parenchyma (**A**). It demonstrates solid growth of mostly eosinophilic cells, admixed with ‘clear’ cells with intracytoplasmic vacuoles (**B**). The eosinophilic cells have voluminous cytoplasm with large intracytoplasmic vacuoles; the nuclei show prominent to often very large nucleoli (**C,D**). Cathepsin K is positive on immunohistochemistry (**E**). On electron microscopy, numerous intracytoplasmic mitochondria are present (**F**).

All sporadic tumors were typically solid (except one case that showed focal gross cysts), small size and low-stage (pT1) [36,37]. The mean tumor size was 3.4 cm (range, 1.5 to 7 cm) [35,36]. The cut surface demonstrated tan/mahogany-brown color, often mimicking the color of the adjacent kidney parenchyma, as often seen in renal oncocytoma, without a gross evidence of necrosis or hemorrhage (Figure 3A).

#### 3.2.2. Microscopic Features

At low power, HOT is a well-circumscribed, but non-encapsulated tumor that demonstrates solid to nested growth with focal tubulocystic features (Figure 3B). Prominent intratumoral vessels of medium to large caliber and rare entrapped renal tubules can be found at the periphery [35]. The nested growth is more typically found in a loose stroma. The cells show voluminous eosinophilic cytoplasm and frequent large intracytoplasmic vacuoles, which can be quite prominent and easily recognizable at low to mid-power (Figure 3B–D) [35]. The nuclei are round to oval with conspicuously enlarged nucleoli that focally mimic viral inclusions and invoke a “high-grade” appearance, which at first impression can be worrisome [35]. Perinuclear halos and nuclear irregularities are typically not found.

#### 3.2.3. Immunohistochemical Features, Electron Microscopy and Molecular Findings

HOT shows frequent reactivity for CD117 with CK7 only focally positive in scattered cells, an immunoprofile mimicking the one typically found in renal oncocytoma (oncocytoma-like immunoprofile) [35,36]. Cathepsin K is, however, invariably positive, either as diffuse or focal (Figure 3E) and CD10 was positive in great majority of reported cases [35]. All cases reported by He et al. were also reactive for PAX8, AE1/AE3, CK18, antimitochondrial antigen, and SDHB, and were negative for vimentin, TFE3, HMB45, and melan-A [35]. None of the evaluated cases showed *TFEB* and *TFE3* gene rearrangements [35].

We have also studied two HOT cases by electron microscopy and both cases demonstrated an ‘oncocytoma-like’ appearances with numerous intracytoplasmic mitochondria (Figure 3F).

Using NGS, Chen et al. found somatic inactivating mutations of *TSC2* or activating mutations of *MTOR* with a loss of chromosome 1 in the evaluated cases, consistent with a hyperactive *MTOR* complex [36]. None of their evaluated patients had a documented TSC [36]. Using array comparative genomic hybridization (aCGH), He et al. found frequent losses of chromosome 1, chromosome 19, and a loss of heterozygosity on 16p11.2-11.1 and 7q31.31 [35]. Of note, some similarities in these molecular findings in HOT, such as losses of *TSC2* and *TSC1* and activation of the *MTOR* pathway have also been found in ESC RCC, another emerging renal entity, covered in this review, that nevertheless shows quite different morphology from HOT [1,2,5,9,10,13].

### 3.3. Differential Diagnosis

The main differential diagnosis for HOT includes renal oncocytoma, Chr RCC and the broad category of “hybrid” oncocytic tumors, seen in the sporadic or syndromic setting.

Although various morphologic patterns and atypical features of renal oncocytoma have been well documented [43], the typical “high-grade” morphology, as seen in HOT, is essentially beyond the permissible morphology for renal oncocytoma, despite the IHC similarities that include the positive CD117, accompanied by CK7 reactivity restricted to rare scattered cells.

Although conspicuous “plant like” growth that is seen in classic Chr RCC can superficially mimic the vacuolated morphology of HOT, Chr RCC lacks marked cytoplasmic vacuoles; importantly, the “high-grade” nuclear features seen in HOT are not part of the Chr RCC morphologic spectrum. Chr RCC also typically exhibits irregular (“raisinoid”) nuclei with perinuclear halos, which are not seen in HOT. On IHC, CK7 is typically diffusely positive in Chr RCC, but CK7 is only reactive in rare cells in HOT. Importantly, cathepsin K and CD10 reactivity seen in HOT are typically absent both in renal oncocytoma and Chr RCC.

The main differential diagnosis for the HOT are the so-called “hybrid tumors” that can be seen either in either a syndromic or sporadic setting [5,38,39,40,41,42]. These “hybrid tumors” are, however, typically multifocal, when encountered in patients with BHD syndrome, renal oncocytosis and TSC. We have also encountered solitary “HOT-like” cases on morphology, that demonstrated *FLCN* mutation (either as a new mutation or in a patient with previously unrecognized BHD), which can be conclusively distinguished from HOT only by molecular analysis (unpublished data).

Other, less common renal tumors that can potentially mimic HOT include MiTF RCC (TFEB and TFE3), oncocytic papillary RCC, SDH-deficient RCC, and ESC RCC and their key distinguishing features are summarized in Table 2.

## 4. Low-Grade Oncocytic Tumor (LOT) of Kidney

Low-grade oncocytic tumor (LOT) has been recently proposed as a distinct renal entity, emerging from the spectrum of oncocytic renal tumors that are difficult to classify [44]. In the only published study to date, consisting of 28 cases collected from four large institutional renal tumor archives, LOT showed fairy consistent morphologic features and immunoprofile characterized by lack of CD117 reactivity and diffuse positivity for CK7 [44]. Although LOT demonstrates an overlapping morphology with renal oncocytoma and eosinophilic Chr RCC, it does not fit completely into either of these entities. Some of these tumors were, and likely still are, labeled using designations such as “eosinophilic Chr RCC”, “oncocytic renal tumor, NOS”, “unclassified/low-grade oncocytic tumor”, “hybrid or hybrid oncocytoma-chromophobe tumor” or “borderline/uncertain/low malignant potential” tumors [38].

To our knowledge, this emerging entity is morphologically similar to the four cases labelled ‘eosinophilic Chr RCC’ that were included in the study by Davis et al., that lacked any copy number alterations; however, no IHC profile was provided for these cases [45]. Another recent study by Ohashi et al. also showed a complete absence of any chromosomal losses in 10/24 (41.7%) of cases designated as “eosinophilic Chr RCC” in a large Japanese-Swiss combined cohort of Chr RCC, whereas only 6/69 (8%) of the “classic Chr RCC” showed absence of any chromosomal loss [46]. The authors pointed out correctly that no stringent diagnostic criteria exist to classify eosinophilic Chr RCC, based on the current 2016 WHO classification, which stipulates that eosinophilic Chr RCC should be “almost purely composed of eosinophilic cells” [47]. Although no overall survival differences were found between the “classic Chr RCC” and the “eosinophilic Chr RCC” cases, their study did not provide more granular outcome data, for example on disease-specific survival or disease-specific progression in these subgroups [46].

LOT is typically a single, small tumor that shows low stage and, based on the available data, it exhibits an indolent clinical behavior [44]. The awareness of the constellation of the clinical, morphologic and immunophenotypic features of this tumor that we provisionally named “LOT” will hopefully increase its recognition and will prompt re-evaluation of similar oncocytic tumors that were diagnosed using various terms.

### 4.1. Clinical Features

LOT is typically a single and sporadic tumor, found in a non-syndromic setting and in older patients (median age 66 years). There is a female predilection (M:F = 1:1.8) [44]. During the follow-up, these tumors behaved indolently, with no evidence of disease progression, although this is based on a single study with a relatively limited follow-up (mean 31.8 months) [44].

### 4.2. Pathological Features

#### 4.2.1. Gross Features

**Figure 4:**
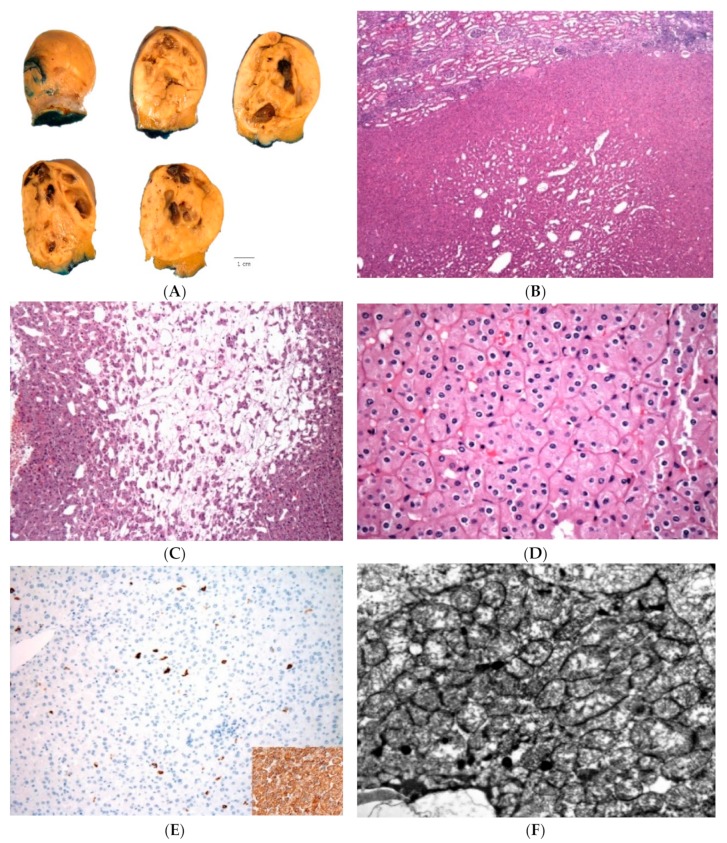
Low-grade oncocytic tumor (LOT) of kidney grossly shows tan to yellow cut surface (**A**). LOT is a non-encapsulated tumor showing mostly solid growth; focal tubular-tubuloreticular patterns can be present more centrally (**B**). There are frequent, sharply delineated edematous areas containing single cells and irregularly cell cords (**C**). The eosinophilic cells show ‘low-grade’, round to oval nuclei, often with delicate perinuclear clearing (**D**). On immunostains, CD117 is typically negative and there is diffuse reactivity for CK7 (insert) (**E**). Electron microscopy shows densely packed intracytoplasmic mitochondria (**F**).

LOT is typically a tan/yellow-brown, solid tumor; some examples showed macrocysts (Figure 4A). Multiple tumors or bilateral kidney involvement have not been documented so far. Median tumor size was 3 cm (range 1.1–13 cm) and the great majority (88%) of tumors were stage pT1a (68%) or pT1b (20%) [44].

#### 4.2.2. Microscopic Features

LOT of kidney is a non-encapsulated neoplasm that exhibits solid, compact nested or focal tubular and tubuloreticular growth, particularly to the more central parts (Figure 4B) [44]. Sharply delineated lose stromal, edematous areas are frequently found, containing cells that show irregular, loose reticular, cord-like and individual cell growth, often resembling disorganized tissue culture (Figure 4C) [44]. Focal lymphocytic clusters or more delicate and irregular intercellular lymphocytic clusters can be often seen in the more solid tumor areas. The cells show homogeneous oncocytic cytoplasm, round to oval nuclei that typically lack significant irregularities and “raisinoid” shapes. The nuclei may focally show delicate perinuclear halos or clearings (Figure 4D). No atypical or potentially worrisome morphologic features were noted, such as significant cell atypia, pleomorphism, mitotic activity or coagulative necrosis.

#### 4.2.3. Immunohistochemical Features, Electron Microscopy, Molecular Features

LOT of kidney is invariably CD117-negative and diffusely CK7-positive, which is a typical and constant immunopattern (Figure 4E) [44]. Of note, rare cases may show only focal and weak CD117 reactivity. LOT is also positive for AE1/AE3, PAX8, E-cadherin, BerEP4 and MOC31; fumarate hydratase is retained [44]. LOT is negative for CA9, CK20, CK5/6, p63, CD15, HMB45, melan-A, and vimentin [44]. CD10 and AMACR are mostly negative or only focally positive. Muller–Mowry colloidal iron staining was either negative or only luminal positive.

On electron microscopy, LOT demonstrates abundant, closely packed intracytoplasmic mitochondria, similar to oncocytoma, which supports the descriptor “oncocytic” for this entity (Figure 4F).

Only limited molecular data are available so far that show no consistent chromosomal losses or gains of multiple chromosomes; in some cases a disomic chromosomal status was found [44]. Evaluated cases showed frequent deletions at 19p13.3, 1p36.33 and 19q13.11 [44].

### 4.3. Differential Diagnosis

The main differential diagnosis for LOT includes renal oncocytoma and Chr RCC. The similarities with oncocytoma include: an absence of capsule, mostly diffuse solid growth, cells with uniform oncocytic cytoplasm and typically round to oval nuclei. In contrast to oncocytoma, LOT has areas of edematous stroma that are sharply delineated from the solid areas. These hypocellular areas show disorganized “tissue culture-like” cell arrangement and contain cells forming irregular cords and single cells. These areas are somewhat different from the typical “archipelaginous” areas that are often found in the more central parts of the renal oncocytoma. The cell of renal oncocytoma, in contrast to LOT, also lack perinuclear halos, and are usually diffusely reactive for CD117, whereas CK7 stains only scattered cells.

LOT exhibits as well some similarities with the eosinophilic variant of Chr RCC that include: absence of a peripheral capsule, presence of diffuse and solid growth, cells with perinuclear clearings (halos), and diffuse CK7 reactivity. In contrast to LOT, eosinophilic Chr RCC typically lacks the focal edematous and hypocellular areas; the cells exhibit more prominent membranes, and the nuclei demonstrate more irregular shapes and perinuclear halos. CD117 is also diffusely positive in great majority of Chr RCC, including the eosinophilic Chr RCC.

An extended differential diagnosis that includes other renal tumors with oncocytic or eosinophilic features include several additional entities, which are usually easily distinguished from the core group of renal “oncocytic” tumors, that include oncocytoma, eosinophilic Chr RCC and the broader group of “borderline/hybrid” oncocytic tumors. A summary of the features that are helpful in distinguishing LOT from the other kidney tumors with oncocytic/eosinophilic cytoplasm is shown in Table 2.

## 5. Conclusions

In this review we summarized the current knowledge and available data on four emerging, provisional renal entities, that include ALK-RCC, which is already included in the WHO 2016 classification as an ‘emerging/provisional’ entity, as well as three previously unrecognized entities that are not currently included in the WHO classification, eosinophilic solid and cystic renal cell carcinoma (ESC RCC), low-grade oncocytic renal tumor (LOT) and high-grade oncocytic renal tumor (HOT) of kidney. Importantly, the recognition of these novel entities rests primarily on their distinct morphologic features with the aid of IHC. We hope that this review will promote an awareness of these emerging renal tumor entities and additional studies for their further evaluation and characterization.

## Figures and Tables

**Table 1:** Summary of the features of emerging renal tumors eosinophilic solid and cystic renal cell carcinoma (ESC RCC), anaplastic lymphoma kinase rearrangement-associated RCC (ALK-RCC), low-grade oncocytic renal tumor (LOT) and high-grade oncocytic renal tumor (HOT).

Emerging Renal Tumor	Clinical Features	Morphology	Immunohistochemistry	Molecular Features	Prognosis
Eosinophilic solid and cystic RCC (ESC RCC)	Mostly females and solitary tumors, ~10% in TSC patients	Solid and cystic growth, often scattered histiocytes and lymphocytes, voluminous eosinophilic cells, cytoplasmic coarse granularity (stippling)	CK20+ either focal or diffuse (note: 10%–15% CK20-), CK7-, CD117-	Somatic bi-allelic loss or mutation of *TSC1* and *TSC2*	Typically good, but rare cases documented with adverse prognosis
Anaplastic lymphoma kinase rearrangement-associated RCC (ALK-RCC)	Adults (younger middle age or older); children or adolescent with the sickle cell trait	Variable and admixed morphologies in adults, often mucinous background present; renal medullary carcinoma-like morphology in children	ALK1+, remaining IHC nonspecific; rare cases in children TFE3+ (but without translocation by FISH)	*ALK* rearrangement with various fusion partners: *VCL*, *TPM3*, *EML4*, *STRN*, *HOOK1*, *CLIP1* and *KIF5 B*	~1/3 adverse prognosis (metastatic disease at presentation)
High-grade oncocytic tumor (HOT)	Wide age range, female/male = 4/1; can occur rarely in TSC patients; typically smaller tumors	Solid growth, oncocytic/eosinophilic cells, prominent cytoplasmic vacuoles round to oval nuclei, often very large nucleoli	Cathepsin K+, CD10+, CD117+, CK7- (only rare scattered cells +)	Limited data: non-overlapping mutations in either *TSC1*, or *TSC2*, or *MTOR* genes; by aCGH loss of 19 p or chr. 1	Indolent tumors (limited follow-up)
Low-grade oncocytic tumor (LOT)	Older patients, solitary and non-syndromic tumors, small size	Solid growth with sharp transition to edematous areas with loose cell arrangement; low-grade oncocytic cells, round to oval nuclei and frequent perinuclear halos	CD117- (rarely very weak+), CK7+	Limited data: by aCGH deletions at 19p13.3, 1 p36.33 and 19q13; also disomic status in some	Indolent tumors (limited follow up)

**Table 2:** Key distinguishing features of the emerging renal tumors vs. other eosinophilic/oncocytic renal tumors.

Diagnosis	Key Distinguishing Features	Immunohistochemistry
Eosinophilic, solid and cystic RCC (ESC RCC)	Great majority females, solid and cystic growth, cytoplasmic stippling, lacks perinuclear halos	CK20+ (diffuse or focal; rarely CK20-), CK7-, CD117-
Anaplastic lymphoma kinase rearrangement-associated RCC (ALK RCC)	Variable and mixed morphology in adults, often mucinous background. Renal medullary carcinoma-like morphology in children	ALK1+, rare TFE3+ (FISH-)
High-grade oncocytic tumor (HOT)	Solid growth, voluminous oncocytic cells with high grade nuclei, large cytoplasmic vacuoles	CD117+, CK7- (only scattered cells CK7+), Cathepsin K+, CD10+
Low-grade oncocytic tumor(LOT)	Solid growth with gradual transition to trabecular areas; sharply delineated edematous stromal areas with loose and irregular cell arrangement	CD117-, CK7+ (diffuse)
Oncocytoma	Solid growth at the periphery, can show tubulocystic growth, central ‘archipelaginous’ areas, cells lack perinuclear halos	CD117+, CK7- (usually only scattered cells CK7+)
Chromophobe RCC, eosinophilic	Solid growth, loose stromal areas absent, cells with more prominent membranes, irregular (‘raisinoid’) nuclei, perinuclear halos	CD117+, CK7+
Clear cell RCC, eosinophilic	At least focal clear cell areas, delicate vasculature in the background	CA9+, CD117-
Papillary RCC, oncocytic/solid	Papillary growth (at least focally)	AMACR+, CK7+, CD10+
Epithelioid angiomyolipoma	Epithelioid cells, may be pleomorphic, lacks perinuclear halos	PAX8-, Cathepsin K+, HMB45+, AE1/AE3-
SDH-deficient RCC	Low-grade oncocytic cells with at least focal flocculent (fluffy) cytoplasm and inclusions; lacks perinuclear halos	CD117-, SDHB-, AE1/AE3- (often)
